# The Potential Benefits of Using Garlic Oil and Its Active Constituent, Dially Disulphide, in Combination With Carvedilol in Ameliorating Isoprenaline-Induced Cardiac Damage in Rats

**DOI:** 10.3389/fphar.2021.739758

**Published:** 2021-09-27

**Authors:** Syed Mohammed Basheeruddin Asdaq, Obulesu Challa, Abdulhakeem S. Alamri, Walaa F. Alsanie, Majid Alhomrani, Mohammed Asad

**Affiliations:** ^1^ Department of Pharmacy Practice, College of Pharmacy, AlMaarefa University, Riyadh, Saudi Arabia; ^2^ Department of Pharmacology, Krupanidhi College of Pharmacy, Bangalore, India; ^3^ Department of Clinical Laboratory Sciences, the Faculty of Applied Medical Sciences, Taif University, Taif, Saudi Arabia; ^4^ Centre of Biomedical Sciences Research (CBSR), Deanship of Scientific Research, Taif University, Taif, Saudi Arabia; ^5^ College of Applied Medical Sciences, Shaqra University, Shaqra, Saudi Arabia

**Keywords:** GC-MS, antioxidants, garlic oil, diallyl disulphide, herbal supplements, carvedilol cellular damage

## Abstract

Garlic oil and its primary component, diallyl disulphide (DADS), were tested in rats with isoprenaline (ISO) induced myocardial infarction for cardioprotective benefits when combined with carvedilol. Garlic oil (GO) was administered to rats (Sprague-dawley strain) at two doses of 50 and 100 mg/kg body weight, whereas DADS was given in two doses of 4.47 and 8.94 mg/kg, respectively. The animals were given oral doses of garlic oil and DADS on alternate days for 3 weeks, either alone or in combination with carvedilol (2 mg/kg). Cardiac injury was done by administering two doses of isoprenaline (150 mg/kg, sc) to all treated groups except the first, which served as a control. Biomarkers of cardiac injury and histological investigations were studied for their potential in reducing ISO-induced myocardial damage. Animals pretreated with GO, DADS, and carvedilol had significantly (*p* < 0.01) lowered heart weight and heart to body weight ratio. In rats treated with carvedilol plus high dosages of garlic oil (100 mg/kg, p.o) and DADS (8.94 mg/kg, p.o) compared to the ISO control and carvedilol group, the activities of SOD and Catalase were enhanced in cardiac tissue homogenate. When compared to ISO control and carvedilol group, the activities of LDH and CK-MB were elevated in heart tissue homogenate with a simultaneous reduction in their serum levels in animals treated with a combination of carvedilol with high doses of garlic oil (100 mg/kg, p.o) and DADS (8.94 mg/kg, p.o). Overall, combining garlic oil or DADS with carvedilol improved the cardioprotective effect of carvedilol and protected rats from ISO-induced myocardial infarction. However, more research is needed to establish the mechanism of garlic oil and DADS interaction with carvedilol.

## Introduction

Consumption of herbal medicines or nutritional supplements along with modern medicine is a common practice throughout the world. This is done with the belief that the addition of herb(s) or nutrient(s) to drug regimen may provide extra beneficial effects and/or reduce adverse effects of the drugs (Shaikh et al., 2020). The effect of many herbs and nutrients on the pharmacological effects of drugs is unknown, though some herbs and nutrients have been reported to affect the overall pharmacological effects of drugs due to pharmacodynamic and/or pharmacokinetic interactions (Ronis et al., 2018).

Myocardial infarction (MI), generally known as a heart attack, is one of the most dreadful disorders. According to estimates, MI will kill approximately 23.3 million individuals by 2030. Mortality due to MI is increasing at an alarming rate in Asian countries such as India and Malaysia (Xu et al., 2016; Rosello et al., 2017). Atherosclerosis is the most prevalent cause of MI, which generates a discrepancy in production and consumption in the myocardium, leading to hypoxic and waste product accumulation, that eventually leads to mycoyte mortality (Kumar et al., 2016). Despite this, the pathophysiology of MI remains a mystery. Inflammation and necrosis, on the other hand, have been identified as key factors in MI in a number of investigations (Goyal et al., 2015; Othman et al., 2017).

Isoprenaline (ISO) is a non-selective agonist for β adrenergic receptors. Administration of ISO at a higher concentration leads to a fall in myocardial compliance due to ischemic damage, and it is one of the most widely used models for assessing the cardioprotective efficacy of new drugs and studying the pathological consequences of human myocardial impairment (Zhang et al., 2005).

Garlic bulbs from the plant *Allium sativum* (family- Amaryllidaceae) are common flavouring agent added to different food recipes. Garlic is also known to possess excellent medicinal properties. The garlic bulb and its different preparations, such as garlic oil, garlic powder, and various garlic extracts, are mentioned for their therapeutic benefits in different traditional systems of medicine (Adaki et al., 2014). Further, the pharmacological and therapeutic properties of garlic and its constituents have been investigated by several authors earlier (Chang et al., 2011; Lissiman et al., 2014; Hou et al., 2015; Nicastro et al., 2015; El-Saber Batiha et al., 2015). We have earlier reported cardiovascular actions and interactions of garlic and its different preparations with several drugs. Garlic showed antihypertensive effect and enhanced bioavailability and antihypertensive effects of propranolol and hydrochlorthiazide in our earlier studies (Asdaq and Inamdar, 2009; Asdaq and Inamdar, 2011a; Asdaq and Inamdar, 2011b). Garlic and its active constituent s-allyl cysteine also enhanced the cardioprotective and antihypertensive effects of captopril (Asdaq and Inamdar, 2010). We also reported that garlic, aged garlic extract and s-allyl cysteine showed antioxidant and hypolipidemic effects that were influenced by the administration of conventional antihypertensives (Asdaq et al., 2009b; Asdaq, 2015). Recently, we reported the interaction of aged garlic extract and its constituent s-allyl csyteine on isoprenaline induced myocardial infarction in rats (Asdaq et al., 2021).

The active constituents of garlic and the most effective garlic preparation with cardiovascular benefits are not correctly known. Different studies have given conflicting reports on the active constituents and the best garlic preparation for cardiovascular benefits. Garlic constituents with potent antioxidant action such as s-allylcysteine and s-allylmercaptocysteine are reported as the constituents responsible for the beneficial effect of garlic on the cardiovascular system. Earlier, allicin (allyl 2-propenethiosulfinate) was believed to be the constituent with a cardioprotective effect, but it is a highly unstable compound and it gets converted to s-allylcysteine and s-allylmercaptocysteine in the body. Garlic oil is a typical garlic product that has been shown to improve anti-oxidant enzyme activity (Ko et al., 2016). Active components of garlic oil, such as diallyl sulphide (DAS), and diallyl disulphide (DADS), have been found to protect and treat oxidative damage (Guan et al., 2018). Garlic oil has been demonstrated to aid weight loss by reducing LDL cholesterol levels (Yang et al., 2018). Because hyperlipidemia is a known cause of MI, we want to learn more about the role of garlic oil in preventing ischemia damage, as well as whether its main active ingredient, diallyl disulphide (DADS), has similar or different cardioprotective properties.

Beta-adrenergic blockers are a class of drugs with multiple pharmacological actions. Carvedilol is a non-selective beta-blocker that also blocks alpha_1_-adrenergic receptors, providing cardioprotective action with vasodilation. It is used in the treatment of hypertension, angina pectoris, cardiac arrhythmias, and also as an antioxidant and antiproliferative agent. Carvedilol is used in the treatment of left ventricular dysfunction and congestive heart failure. Apart from these, it also has a lipid lowering effect and augments renal dysfunction, indicating wide spread use of this non-selective beta-blocker (Singh and Preuss, 2021).

Continuing our efforts to determine the beneficial effects of garlic preparations and their influence on the effects of drugs affecting cardiovascular functions, the present study determined the effect of garlic oil and diallyl disulphide (DADS) on the cardioprotective effect of carvedilol on isoprenaline induced myocardial infarction in rats.

## Materials and Methods

### Experimental Animals

Female Sprague-Dawley rats weighing 150–200 g were housed at 25 ± 5 °C in a well-ventilated animal house under 12:12 h light dark cycle. The rats had free access to standard rat chow (Amrut Laboratory Animal Feed, Maharashtra, India) containing (% w/w) protein 22.10, oil 4.13, fiber 3.15, ash 5.15, sand (silica) 1.12 and water *ad libitum*. The institutional animal ethics committee of Krupanidhi College of Pharmacy (KCP/IAEC-27) approved the experimental protocol and animals were maintained under standard conditions in an animal house approved by Committee for the Purpose of Control and Supervision on Experiments on Animals (CPCSEA).

### Materials

Sigma Aldrich (United States) provided the garlic oil, which was GC-MS standardized for the presence of DADS. The CK-MB and LDH kits were provided by Crest Biosystems and Coral Clinical Systems in Goa, India. All of the compounds utilized in this investigation were analytical grade and came from a standard source.

### Quantification of DADS in Garlic Oil by GC-MS

A Hewlett Packard 5890 II GC with an HP 5972 Mass selective detector and HP-5ms capillary column (30 m 0.25 mm, film thickness 0.25 m) was used to assay the garlic oil sample for DADS. The injector and detector were adjusted to 220 and 290°C, respectively. The temperature of the column was raised from 50 to 220°C at a rate of 3°C/min for 10 min. At a flow rate of 1 ml/min, helium was used as the carrier gas. In the splitless method, 1 μL (1.0 L) of the concentrate was manually injected into 10 ml of each organic extract. An electron ionization device with a 70 eV ionization energy was employed for GC/MS detection. Temperatures for the injector and MS transfer line were set at 220 and 290°C, respectively. The chemicals were tentatively identified by comparing their mass spectra to those of the NIST 98 and Wiley 275 library data of the GC-MS system (Kimbaris et al., 2006).

### Dose Selection

**FIGURE 1:**
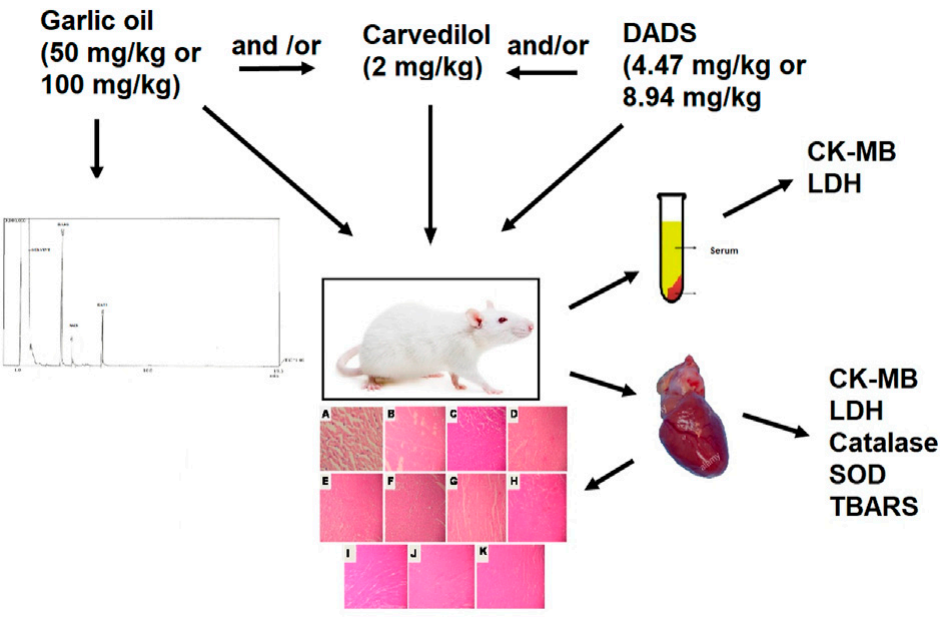
Schematic representation of procedure followed.

**FIGURE 2:**
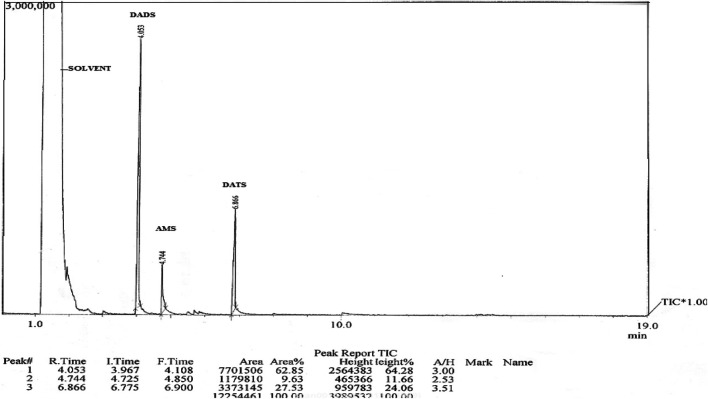
GC-MS profile of garlic oil The GC-MS profile of garlic oil shows different peaks. Peak observed at retention time (RT) of 1 min represents the solvent. The DADS was observed at an RT of 4 min while peaks for allyl methyl sulphide (AMS) and diallyl trisulphide (DATS) was observed at 5 and 7 min respectively.

Garlic Oil (50/100 mg/kg, *p.o*) (Kuo et al., 2011) and carvedilol (2 mg/kg, *p.o*) (Watanabe et al., 2002) doses were selected from previous studies. The required doses of DADS (4.47/8.94 mg/kg) were selected based on GC-MS peaks of garlic oil (Figure 1 and Figure 2) Experimental protocol.

### Experimental Protocol

There were eleven groups of experimental animals used in this study, each having six rats. Group I was kept as normal control and given a vehicle (1 ml/kg, *p.o*) on alternate days for 3 weeks. The vehicle used was acacia (2% w/v). Groups 2 to 11 received the following treatments on alternate days for 3 weeks, followed by administration of two doses of isoprenaline (ISO) at a dose of 150 mg/kg subcutaenously for two consecutive days (Pipaliya et al., 2012). Group 2 was named ISO control and received vehicle (1 ml/kg, *p.o*) while Group 3 was administered with carvedilol (2 mg/kg, *p.o*). Groups 4 and 5 received garlic oil orally at a dose of 50 mg/kg (low dose) and 100 mg/kg (high dose) respectively, while groups 6 and 7 received a combination of carvedilol with a low dose and high dose of garlic oil respectively. Similarly, groups 8 and 9 received a low dose of DADS (4.47 mg/kg) and a high dose of DADS (8.94 mg/kg) and groups 10 and 11 received a combination of carvedilol with a low dose of DADS and a high dose of DADS respectively. A schematic diagram of the procedure is given in Figure 1.

As mentioned above, animals in all groups except group-1 received ISO (150 mg/kg, s.c) for two consecutive days. After 48 h of the first dose of ISO, blood was withdrawn from animals under anesthesia induced by a combination of ketamine hydrochloride (75 mg/kg, i.p) and xylazine (10 mg/kg, i.p) (Wellington et al., 2013). Serum levels of lactate dehydrogenase (LDH), and creatinine kinase-MB (CK-MB) were determined using commercially available biochemical kits. Following blood removal, a thoracic incision was performed, and the hearts were split open, flushed with saltwater (0.9 percent NaCl), and dried. The weight of the heart was assessed (Buerke et al., 1998) and heart tissue homogenate (HTH) was prepared separately for three hearts in an ice-cold 0.25 M sucrose solution using a mortar and pestle. The homogenate was then centrifuged at 5000 rpm for 15 min. After draining the supernatant, biochemical and molecular studies were performed (Lee et al., 2012). The LDH and CK-MB were estimated using commercially available kits. The estimation of superoxide dismutase (SOD) (Elstner and Heupel, 1976), catalase (Link, 1988) and thiobarbituric acid reactive substances (TBARS) (Niehaus and Samuelsson, 1968) were done in heart tissue homogenate. Slides for microscopic examination were made from the remaining hearts of three animals in each group for histological studies. Five micrometer sections were cut and stained using H and E stain. Depending on the severity of cardiac damage, scores were assigned between 0 and 3 with 0 being no damage, one- mild damage as indicated by degenerations at different foci and slight inflammation. A score of two was given when severe degeneration of myofibrils was observed with or without diffuse inflammation, while a score of three was assigned to slides that showed necrosis along with diffuse inflammation (Karthikeyan et al., 2007).

### Data Analysis

One-way analysis of variance (ANOVA) was used to determine statistical significance, followed by Tukey’s multiple comparison tests using the GraphPad Prism 8.0 computer software kit. The data was presented as mean ± SEM, with a *p* < 0.05 significance level chosen. Power analysis was carried out using G power application, keeping 0.05 as the alpha error probability in One-was ANOVA and post-hoc tests for parameters with low sample size (n = 3).

## Results

### GC-MS Analysis of DADS in Garlic Oil

**FIGURE 3:**
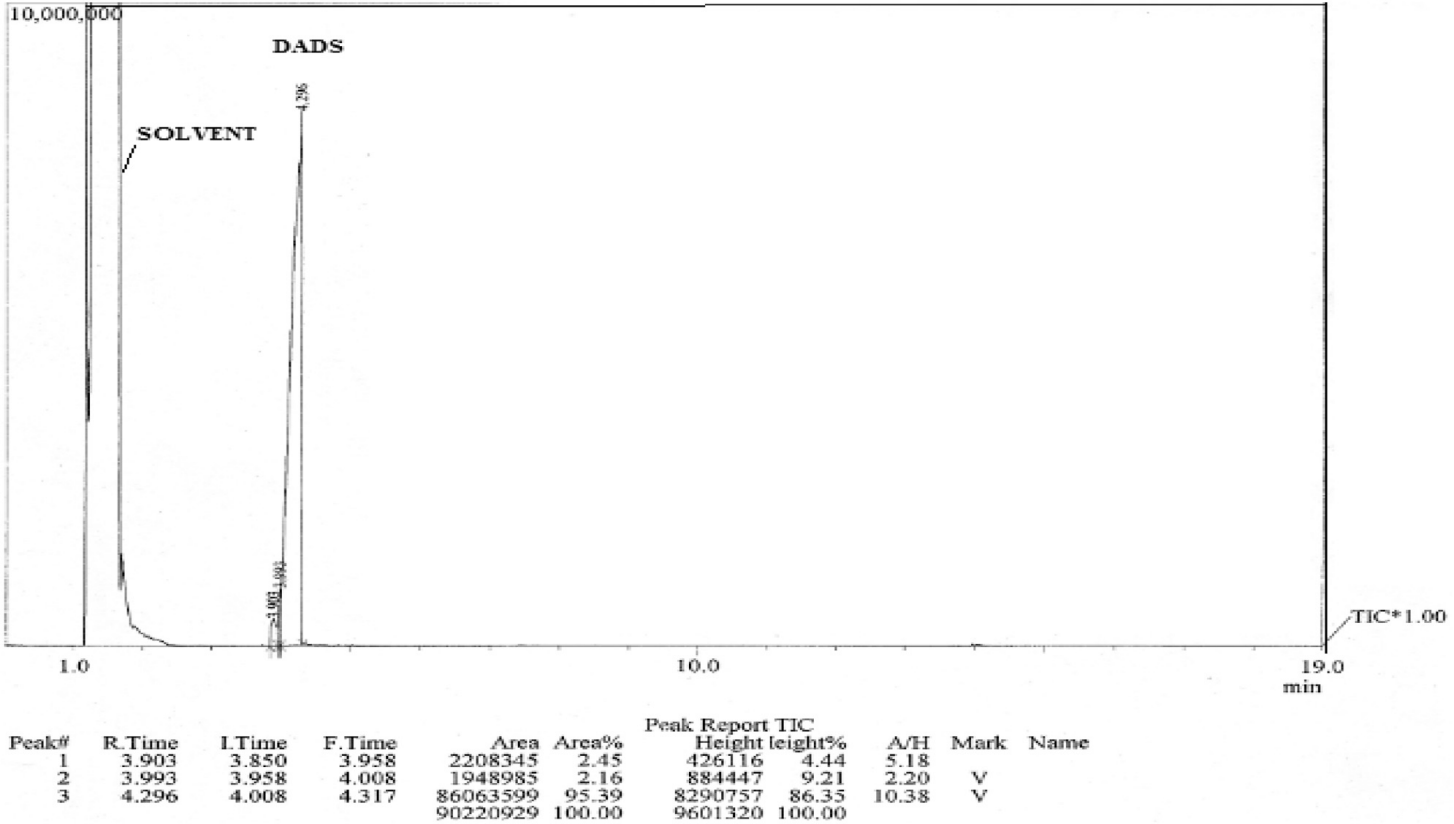
GC-MS profile of diallyl disulphide The GC-MS profile of DADS shows two peaks. At an RT of 1 min, a peak for solvent was observed and DADS peak was observed at an RT of 4 min.

Analysis of garlic oil showed that it contained 8.94% w/w of DADS. On the basis of GC-MS peaks of garlic oil, the DADS doses were determined as 4.47 and 8.94 mg/kg (Figure 2 and Figure 3). The other compounds detected in the oil were allyl methyl sulphide (AMS) and diallyl trisulphide (DATS).

### Effect on Heart Weight and Heart to Body Weight Ratio

The heart weight and the ratio of heart weight to body weight at the end of treatment are shown in Table 1. When compared to the normal control group, animals given two doses of isoprenaline (isoprenaline control) had a substantial (*p* < 0.01) increase in heart weight and heart weight to body weight ratio. When compared to the isoprenaline control, all animals pretreated with garlic oil, diallyl disulphide, and carvedilol had significantly (*p* < 0.01) lower heart weight and heart to body weight ratio. Moreover, animals given garlic oil/diallyl disulphide plus carvedilol had a substantial (*p* < 0.05) improvement in heart weight and heart weight to body weight ratio as compared to the garlic oil and diallyl disulphide groups, respectively. The heart to body weight ratio was more towards the normal value with high doses of garlic oil and diallyl disulphide than with their respective low doses.

**TABLE 1:** Effect on heart weight and heart/body weight ratio.

Groups	Heart weight (g)	Body weight (g)	Heart to body weight ratio (percentage)
Normal control	0.422 ± 0.06	177.34±1.31	0.237 ± 0.01
ISO control	0.690 ± 0.07^***^	172.32±4.81	0.400 ± 0.01^***^
Carvedilol	0.472 ± 0.05^●●^	173.56±5.31	0.271 ± 0.01^●●●^
GOLD	0.524 ± 0.06^●●^	171.45±6.21	0.305 ± 0.01^●●^
GOHD	0.484 ± 0.08^●●^	173.55±7.21	0.278 ± 0.01^●●^
DADSLD	0.532 ± 0.07^●●^	174.21±8.36	0.305 ± 0.01^●●^
DADSHD	0.498 ± 0.10^●●^	172.28±9.36	0.289 ± 0.01^●●^
GOLD + CAR	0.447 ± 0.08^●●●▫^	175.89±1.69	0.254 ± 0.01^●●●▫^
GOHD + CAR	0.436 ± 0.11^●●●▫^	174.29±2.39	0.250 ± 0.01^●●●▫^
DADSLD + CAR	0.431 ± 0.04^●●●▫^	173.37±4.55	0.248 ± 0.01^●●●▫^
DADSHD + CAR	0.426 ± 0.12^●●●▫^	172.89±4.35	0.246 ± 0.01^●●●▫^

Values are given as mean ± Standard error of mean for six rats in each group; ^***^ *p* < 0.001 when compared to normal control; ^●●^ *p* < 0.01, ^●●●^ *p* < 0.001 compared to Isoprenaline control; ^▫^ *p* < 0.05, compared to Garlic oil/Diallyl dilsulphide respective groups. CAR: Carvedilol (2 mg/kg, *p.o*); GOLD: Garlic oil (50 mg/kg, *p.o*); GOHD: Garlic oil (100 mg/kg, *p.o*); DADSLD: diallyl disulphide (4.47 mg/kg, *p.o*); DADSHD: diallyl disulphide (8.94 mg/kg, *p.o*).

### Effect on Biomarkers of Cardiac Damage

The administration of ISO produced significant changes in different biomarkers of cardiac damage. There was no mortality in animals treated with ISO.

#### Effect of CK-MB Activity

**FIGURE 4:**
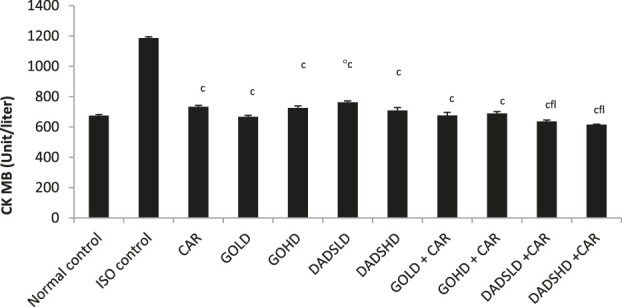
Effect on serum CK-MB levels All values are mean ± SEM, n = 6, ^°^ *p* < 0.01when compared to normal control; ^c^P <0.001 compared to ISO control^f^P<0.001compared to carvedilol; ^l^P<0.001 when compared to respective DADS dose; CAR: Carvedilol (2 mg/kg, *p.o*); GOLD: Garlic oil (50 mg/kg, *p.o*); GOHD: Garlic oil (100 mg/kg, *p.o*); DADSLD: diallyl disulphide (4.47 mg/kg, *p.o*); DADSHD: diallyl disulphide (8.94 mg/kg, *p.*o).

Administration of ISO induced myocardial infarction as indicated by an increase in cardiac specific serum CK-MB levels and a decrease in CK-MB activity in HTH of ISO treated animals when compared to normal controls (*p* < 0.001). Carvedilol, garlic at both doses and DADS at both doses showed cardioprotective effects. The serum levels of CK-MB were reduced and an increase in CK-MB levels in HTH was observed in these groups when compared to the ISO control. Animals treated with a low dose of DADS (4.47 mg/kg, *p.o*) showed a significant increase in serum CK-MB compared to the normal group. Both doses of DADS in combination with carvedilolproduced a significant fall in serum CK-MB level compared to the carvedilol treated group. Both doses of DADS in combination with carvedilolshowed a significantly lower serum CK-MB activity compared to DADS treatment alone (Figure 4).

**FIGURE 5:**
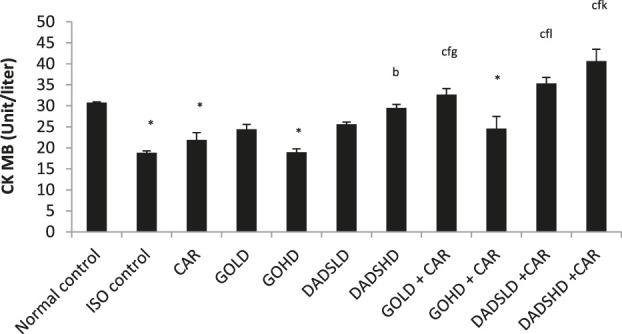
Effect of CK-MB levels in the HTH All values are mean ± SEM, n = 6, ^*^ *p* < 0.001 when compared to normal control; ^b^P<0.01, ^c^P <0.001 compared to ISO control; ^d^P<0.05, ^f^P<0.001compared to carvedilol; ^g^P <0.05 when compared to respective garlic oil dose; ^j^P<0.05, ^k^P<0.01, ^l^P<0.001 when compared to respective DADS dose; CAR: Carvedilol (2 mg/kg, *p.o*); GOLD: Garlic oil (50 mg/kg, *p.o*); GOHD: Garlic oil (100 mg/kg, *p.o*); DADSLD: diallyl disulphide (4.47 mg/kg, *p.o*); DADSHD: diallyl disulphide (8.94 mg/kg, *p.o*).

In HTH, animals treated with a high dose of garlic oil (100 mg/kg, *p.o*) showed a significant decrease in the CK-MB while animals treated with a combination of high dose of DADS (8.94 mg/kg, *p.*o) and carvedilol showed a moderately significant increase in the CK-MB, but the group treated with carvedilol group produced a moderate fall of CK-MB than normal. All treated groups except the low dose of garlic oil (50 mg/kg, *p.o*) and the combination of both doses of DADS with carvedilol showed a significant fall in the CK-MB level in HTH as compared to the ISO group. Furthermore, in groups treated with the low dose of garlic oil (50 mg/kg, *p.o*) and the combination of both doses of DADS with carvedilol, a significant rise in the CK-MB levels was observed compared to the carvedilol alone group. Group treated with a low dose of garlic oil (50 mg/kg, *p.o*) in combination with carvedilol showed a rise in the CK-MB activity compared to low dose of garlic oil (50 mg/kg, *p.o*) alone. Animals treated with a combination of carvedilol with either dose of DADS were observed to have significantly more CK-MB activity compared to either dose of DADS alone (Figure 5).

#### Effect on LDH Activity

**FIGURE 6:**
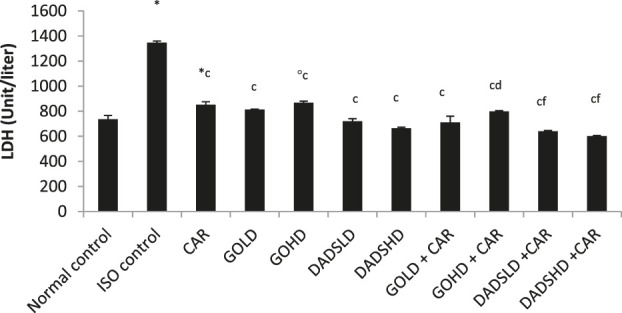
Effect on serum LDH levels All values are mean ± SEM, n = 6, ^°^ *p* < 0.01, ^*^ *p* < 0.001 when compared to normal control; ^c^P <0.001 compared to ISO control; ^d^P<0.05, ^f^P<0.001compared to carvedilol CAR: Carvedilol (2 mg/kg, *p.o*); GOLD: Garlic oil (50 mg/kg, *p.o*); GOHD: Garlic oil (100 mg/kg, *p.o*); DADSLD: diallyl disulphide (4.47 mg/kg, *p.o*); DADSHD: diallyl disulphide (8.94 mg/kg, *p.o*).

**FIGURE 7:**
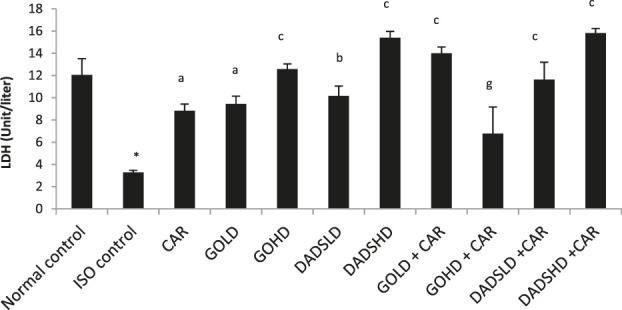
Effect on LDH levels in the HTH All values are mean ± SEM, n = 3, ^*^ *p* < 0.001 when compared to normal control; ^a^P<0.05, ^b^P<0.01, ^c^P <0.001 compared to ISO control; ^g^P <0.05 when compared to respective garlic oil dose; CAR: Carvedilol (2 mg/kg, *p.o*); GOLD: Garlic oil (50 mg/kg, *p.o*); GOHD: Garlic oil (100 mg/kg, *p.o*); DADSLD: diallyl disulphide (4.47 mg/kg, *p.o*); DADSHD: diallyl disulphide (8.94 mg/kg, *p.o*).

Similar to the CK-MB activity, ISO administration for two consecutive days produced an increase in serum levels of LDH and reduced its activity in HTH. However, the activity of LDH was reduced by all the treatments irrespective of dose and combinations when compared to ISO control (Table 1). Animals treated with a higher dose of garlic oil (100 mg/kg, *p.*o) showed a significant increase in the serum LDH, whereas animals treated with DADS (8.94 mg/kg, *p.*o) and carvedilol showed a significant decrease in the serum LDH when compared to normal control animals. In the group of animals treated with a combination of higher dose of garlic oil (100 mg/kg, *p.*o) and carvedilol, a slight decrease in LDH activity was observed, whereas the combination of both doses of DADS with carvedilol showed a significant fall in LDH level compared to the group treated with carvedilol alone. The group of rats treated with a higher dose of garlic oil (100 mg/kg, *p.*o) and carvedilol had significantly higher LDH activity in the HTH compared to the carvedilol treated group. Combined therapy of garlic oil high dose (100 mg/kg, *p.*o) and carvedilol produced a moderately significant increase in LDH in HTH compared to garlic oil high dose (100 mg/kg, *p.*o) alone ((Figure 6 and Figure 7).

### Effect on SOD and Catalase

**FIGURE 8:**
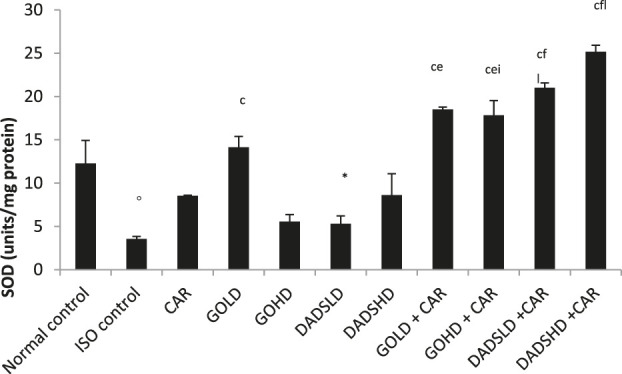
Effect on SOD activity in the HTH All values are mean ± SEM, n = 3, ^°^ *p* < 0.01, ^*^ *p* < 0.001 when compared to normal control; ^c^P <0.001 compared to ISO control; ^e^ *p* < 0.01, ^f^P<0.001compared to carvedilol; ^i^P <0.001 when compared to respective garlic oil dose; ^l^P<0.001 when compared to respective DADS dose; CAR: Carvedilol (2 mg/kg, *p.o*); GOLD: Garlic oil (50 mg/kg, *p.o*); GOHD: Garlic oil (100 mg/kg, *p.o*); DADSLD: diallyl disulphide (4.47 mg/kg, *p.o*); DADSHD: diallyl disulphide (8.94 mg/kg, *p.o*).

The Administration of ISO caused a significant fall in the activities of antioxidant enzymes; SOD and catalase in HTH. Garlic oil (50 mg/kg, *p.*o) and its combination with carvedilol and the combination of both doses of DADS with carvedilol produced a significant increase in the SOD activity compared to the ISO control. Administration of carvedilol along with either dose of garlic oil or DADS was more effective in increasing SOD activity compared to administration of carvedilol alone. The SOD activity was higher in the group treated with a higher dose of garlic oil (100 mg/kg, *p.o*) with carvedilol compared to garlic oil (100 mg/kg, *p.o*) alone. Similarly, the group treated with a combination of carvedilol with DADS (4.47 mg/kg, *p.o*) or DADS (8.94 mg/kg, *p.o*) had significantly higher SOD activity in HTH compared to DADS treatment alone (Figure 8).

**FIGURE 9:**
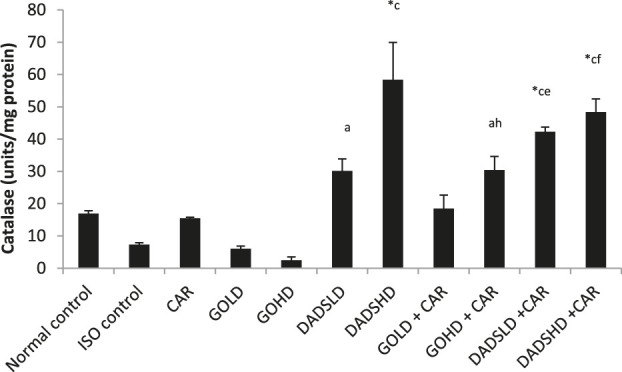
Effect on catalase activity in the HTH All values are mean ± SEM, n = 3, ^*^ *p* < 0.001 when compared to normal control; ^a^P<0.05, ^c^P <0.001 compared to ISO control; ^e^ *p* < 0.01, ^f^P<0.001compared to carvedilol; ^h^ *p* < 0.01 when compared to respective garlic oil dose; CAR: Carvedilol (2 mg/kg, *p.o*); GOLD: Garlic oil (50 mg/kg, *p.o*); GOHD: Garlic oil (100 mg/kg, *p.o*); DADSLD: diallyl disulphide (4.47 mg/kg, *p.o*); DADSHD: diallyl disulphide (8.94 mg/kg, *p.o*).

For catalase activity, the group treated with a high dose of DADS (4.47 mg/kg, *p.o*) in combination with carvedilol showed a moderate increase in catalase activity, while DADS (8.94 mg/kg, *p.o*) treatment alone showed a profound rise in catalase value compared to the normal control group. Animals treated with DADS (8.94 mg/kg, *p.o*) alone and a combination of either dose of DADS with carvedilol produced a significant rise in catalase, whereas DADS (4.47 mg/kg, *p.o*) and garlic oil (100 mg/kg, *p.o*) with carvedilol showed a slight increase in catalase level compared to the ISO group. Both doses of DADS in combination with carvedilol produced a significant rise in catalase levels compared to carvedilol treatment alone. The combination of garlic oil (100 mg/kg, *p.o*) with carvedilol showed a moderate increase in catalase value compared to garlic oil (100 mg/kg, *p.o*) alone (Figure 9).

### Effect on TBARS

**FIGURE 10:**
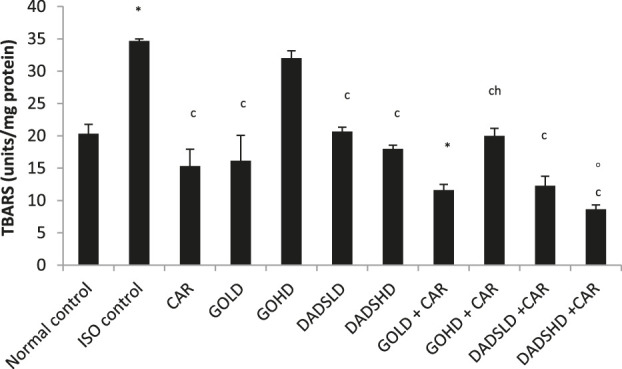
Effect on TBARS in the HTH All values are mean ± SEM, n = 3, ^°^ *p* < 0.01, ^*^ *p* < 0.001 when compared to normal control; ^c^P <0.001 compared to ISO control ^h^ *p* < 0.01when compared to respective garlic oil dose; CAR: Carvedilol (2 mg/kg, *p.o*); GOLD: Garlic oil (50 mg/kg, *p.o*); GOHD: Garlic oil (100 mg/kg, *p.o*); DADSLD: diallyl disulphide (4.47 mg/kg, *p.o*); DADSHD: diallyl disulphide (8.94 mg/kg, *p.o*).

Similar to other biomarkers, the TBARS levels increased significantly upon ISO administration compared to normal control. Animals treated with garlic oil (100 mg/kg, *p.o*) showed a significant increase, whereas those administered DADS (8.94 mg/kg, *p.*o) along with carvedilol showed a moderate decrease in the TBARS compared to normal control. All groups except the high dose of garlic oil (100 mg/kg, *p.o)* produced a significant fall in the TBARS value compared to the ISO control group. The group treated with garlic oil (100 mg/kg, *p.o)* together with carvedilol showed a moderate fall in TBARS value compared to the garlic oil (100 mg/kg, *p.o)* treated group. The combination of DADS (8.94 mg/kg, *p.*o) and carvedilol reduced TBARS values compared to the DADS (8.94 mg/kg, *p.*o) group (Figure 10).

### Effect on Histological Score

**FIGURE 11:**
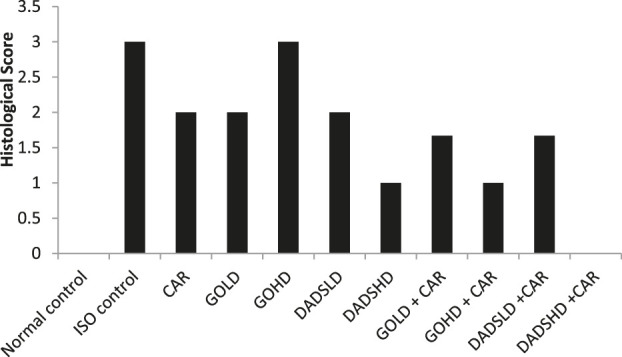
Histological scores of the cardiac tissue.

**FIGURE12:**
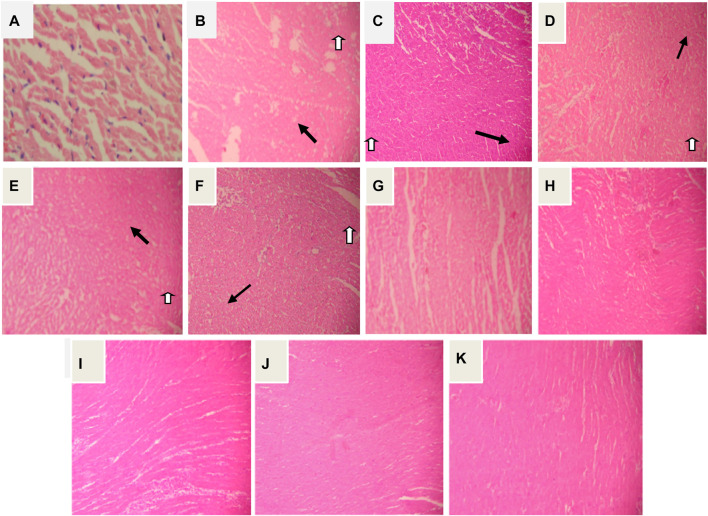
Haematoxylin and eosin (H&E) stained section of heart in isoprenaline induced myocardial damage. Photographed at magnification ×400 **(A)** Heart from normal control group **(B)** Iso control (150 mg/kg, s.c), **(C)** Carvedilol (2 mg/kg) group,**(D)** garlic oil low dose (50 mg/kg), **(E)** garlic oil high dose (100 mg/kg), **(F)** DADS low dose (4.47 mg/kg), **(G)** DADS high dose (8.94 mg/kg), **(H)** garlic oil low dose + CAR, **(I)** garlic oil high dose + CAR, **(J)** DADS low d**o**se + CAR and **(K)** DADS high dose + CAR. A and K are hematoxylin and eosin (400×) stained microscopic sections of Normal control and DADSHD + CAR respectively shown histopathalogical score one i.e. mild necrosis, B and E are hematoxylin and eosin (400×) stained microscopic sections of ISO control and GOHD group respectively have a histopathalogical scoreof four i.e. Severe necrosis, inflammation and fibrosis. C, D and F were hematoxylin and eosin (400×) stained microscopic sections of ATN, GOLD and DADSLD respectively have a histopathalogical scoreof three i.e. Moderate diffuse necrosis, mild inflammation and fibrosis (necrosis shown with bold arrows and fibrosis with hollow arrows). G,H,I and J werehematoxylin and eosin (400×) stained microscopic sections of DADSHD, GOLD + CAR, GOHD + CAR and DADSLD + CAR groups respectively have histopathalogical scoreof 2–2.5 i.e.Mild diffuse, moderate necrosis and mild inflammation.

An average histological score from three animals in each group. Severe necrosis with diffused inflammation was seen after administration of ISO. Pretreatment with DADSHD (8.94 mg/kg, *p.o*) and the combination of both doses of DADS or garlic oil with carvedilol offered protection against ISO induced damage by preventing severe cellular damage. In sections prepared from the hearts of animals of the above mentioned group, only reversible cellular degeneration and slight inflammation were observed. Garlic oil at both doses (50 mg/100 mg/kg, *p.o*) and DADS (4.47 mg/kg, *p.o*) offered less protection against ISO induced cellular damage (Figure 11 and Figure 12).

### Power Analysis

**FIGURE 13:**
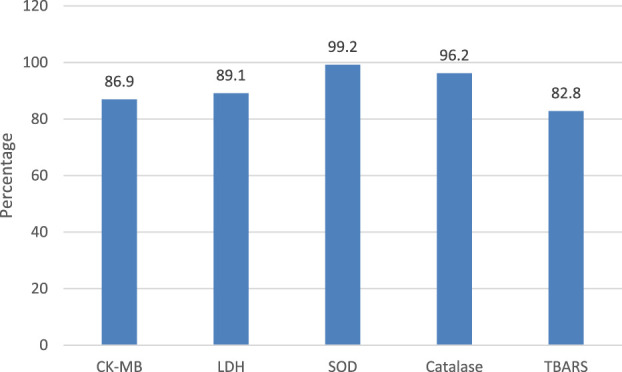
Power Analysis of parameters with low sample size Values are given in percentage, analyzed using G power application, One way ANOVA and Post-hoc test with α error probability of 0.05 and a total sample size of 33 in eleven groups.

Because there were only three samples available for the parameters tested in heart tissue homogenate (HTH), a power analysis was performed to see if the required power of 80% was achieved in all of the observations. The results of the analysis showed 86.9, 89.1, 99.2%, 96.2, and 82.8 percent statistical power to the outcomes obtained in estimates of CK-MB, LDH, SOD, Catalase, and TBARS levels, respectively, because the variance within the group was minimum due to the calibrated experimental set up (Figure 13).

## Discussion

The results of the current study showed varying effects of DADS, garlic oil and their combination with carvedilol on different indicators of cardiac damage in isoprenaline induced myocardial infarction in rats. As mentioned earlier, we have evaluated different preparations of garlic for their effect on the pharmacological activity of commonly used cardiovascular drugs. Our aim has been to confirm the health-promoting effects of garlic supplementation and to identify the best garlic preparation and the chemical constituent(s) responsible for the beneficial cardioavasuclar actions. Since, garlic supplementation is used by many patients using modern medicine, we also determined the interaction between garlic preparations and their active constituents with different drugs.

The present study was done using garlic oil because of earlier reports on the cardiovascular benefits of this oil (Kuo et al., 2011; Seki et al., 2015). The active constituent; DADS, was also selected based on earlier studies on its effect on the cardiovascular system (Khatua et al., 2016) and its dose was selected as equivalent to that present in the garlic oil used in the study through GC-MS analysis.

High doses of isoprenaline (ISO) cause a cascade of events that culminate in ventricular hypertrophy, with disruption and rupturing of the cardiac muscle resulting in extraceullar fluid flow and an increase in heart weight (Liu et al., 2013; Sagor et al., 2015). According to this, in isoprenaline-injected rats, heart weight and the ratio of heart weight to body weight were abnormally high, which was successfully mitigated in rats that received garlic oil, diallyl disulphide, or carvedilol treatment, either separately or jointly. This means that our therapies effectively protect the cardiac muscle against ISO-induced rupture by reducing oxidative stress and limiting water migration to the cardiomyocytes, hence maintaining cardiac homeostasis (Goyal et al., 2010). Carvedilol, like garlic oil and DADS, showed antioxidant properties, albeit the significance of this characteristic is unknown (Ayashi et al., 2016).

The cardiac myocardial infarction was induced using ISO, a catecholamine that increases myocardial function. Administration of ISO for two consecutive days at 150 mg/kg subcutaneously produce severe increase in myocardial function leading to myocardial infarction (Jiang et al., 2019; Feriani et al., 2020). The ISO induced myocardial infarction is one of the most widely used and accepted models of myocardial infarction and is used for evaluation of the cardioprotective effects of various agents. The events of myocardial damage follow the same pattern that is observed with pathological myocardial damage, which starts with biochemical changes followed by ultrastructural to microscopic changes. All these events occur within 48 h after the injection of isoprenaline (Dudnakova et al., 2002). The myocardial damage is also mediated through the generation of oxygen free radicals due to oxidation of ISO, leading to the formation of superoxide anions that ultimately result in the formation of hydrogen peroxide. These oxidative species increase the permeability of microsomes and calcium uptake in the mitochondria. They also reduce ATP formation and damage cellular proteins, lipids, and DNA (Jiang et al., 2019; Feriani et al., 2020). To assess the protection offered by pretreatment of garlic oil, DADS, carvedilol and their combinations, biochemical estimation of enzymes released from cardiac cells; CK-MB and LDH, levels of antioxidant enzymes; SOD and catalase, level of TBARS and histological scores were determined. Elevated serum levels of CK-MB and LDH with a simultaneous decrease in these enzymes in HTH indicates damage to the myocardium. The toxic free radicals and other reactive oxygen species (ROS) are scavenged by endogenous antioxidant enzymes, which include SOD that dismutates superoxide anion and catalase that reduces peroxides (Borek, 2001). Higher enzyme activities suggest enhanced protection against oxidative damage.

Garlic and its different preparations are used traditionally for the treatment of various diseases. Garlic preparations may be classified as allicin-rich and non-allicin preparations (Haina Wang et al., 2013). Allicin-rich preparations are made using raw garlic, while processed garlic is used for the preparation of non-allcin products (Sharifi-Rad et al., 2019). Other than allicin, these two preparations contain several other constituents that are different from each other (Kasuga et al., 2001). Of these different chemicals, organosulfur compounds (OSCs) such as DADS and dially trisulfidehave been reported for several pharmacological actions, including cardiovasulcular effects (Abe et al., 2019). These compounds are also known to modulate activities of different drug metabolism enzymes, especially those involved in phase II metabolism by unknown mechanisms (Zhao et al., 2013).

Carvedilol, a beta-adrenergic blocker, is used as an antianginal, antiarrhythmic, and antihypertentve agent. It is also used for treating both idiopathic and ischemic congestive heart failure. It prevents ISO induced myocardial infarction by antagonising the effect of ISO on the cardiac beta_1_-adrenergic receptors (Elshourbagy, 2016). Carvedilol also displays antioxidant action, although the relevance of this property remains uncertain (Ayashi et al., 2016). However, antioxidant properties that some of these compounds appear to possess have previously been linked to a number of the positive cardiovascular benefits that this group of compounds has been linked to in the literature (Mak and Weglicki, 1988). The findings of this study corroborate prior observations with atenolol, another beta blocker (Asdaq and Avula, 2015). Garlic oil and DADS improved both atenolol and carvedilol’s cardioprotective capacity during ISO-induced myocardial stress in experimental rats. Both beta blockers effects are well-known and have been proven in prior studies (Jonsson et al., 2005).

The garlic oil contains soluble organosulphur compounds called allyl sulphides, which imparts characteristic flavour to the oil. Diallyl disulphide (DADS) is a type of allyl sulphide that is found in garlic oil but is not found in garlic cloves. As mentioned above, DADS is reported to possess several pharmacological activities. It is formed as a product of allicin by the action of the allicinase enzyme during garlic cutting or crushing (Abe et al., 2019).

The ISO administration also produced an increase in the TBARS in the cardiac cells. Antioxidants reduce oxidative stress and reduce both the initiation and propagation of the lipid peroxidation process. Administration of carvedilol along with high dose garlic oil (100 mg/kg, *p.o*) or DADS (8.94 mg/kg, *p.*o) showed a slight fall in TBARS compared to ISO control, indicating a cardioprotective effect. To ascertain the extent of damage to myocardium, a histological examination was carried out along with biochemical estimations. Pretreatment with high doses of garlic oil and DADS alone or with carvedilol substantially maintained the myocardial cellular integrity and decreased the pathological scores when compared to the ISO treated group. The number of samples for determination of different parameters in the HTH was only three. The sample size could not be increased due to ethical issues. Though the sample size was small, it was enough to draw conclusions as the LDH and CK-MB levels were simultaneously determined in the serum.

The results of the present study showed varying effects. Nevertheless, the results suggest that garlic oil or DADS at two different doses augment the cardioprotective action of carvedilol. A comparison of different groups showed a dose dependent effect of garlic oil and DADS, though a significant effect was not observed between the effects produced by the lower dose compared to the higher dose. The comparison of effects produced by the combination of carvedilol with either dose of garlic oil or DADS with individual treatments showed varying effects, with some of them being significant, while many other comparisons were not significant.

The antioxidant property of garlic oil and its constituent diallyl disulphide have been reported earlier (Chiang et al., 2006). The antioxidant action is due to organosulphurous compounds. but the exact mechanism is still not clear. It is apparent that allyl sulphur constituents of these preparations are responsible for accelerated antioxidant enzyme synthesis at times of ISO induced stress to the myocardium (Kim et al., 1997).

## Conclusion

The findings of the present study showed a dose dependent increase in the cardioprotective efficacy of garlic oil and its active constituent, diallyl disulphide (DADS), during isoprenaline induced myocardial damage. Additionally, this study reiterates the role of garlic oil and DADS in amplifying the cardioprotective efficacy of carvedilol in ISO induced ischemic damage in experimental animals. Despite the fact that beta blockers are well-known for their cardioprotective properties, this work was the first to find the cardioprotective interaction of carvedilol with garlic oil and DADS during myocardial stress in rats. The findings of this study will pave the way for further research into the role of garlic oil and DADS in the therapeutic regimen, potentially lowering the dosage of traditional cardioprotective substances like carvedilol.

## Data Availability

The original contributions presented in the study are included in the article/supplementary material, further inquiries can be directed to the corresponding author.
